# Rapid LC–MS Quantification of mRNA Vaccine Capping Efficiency via High-Specificity RNase H Cleavage and Metal Adduct Suppressed Chromatography

**DOI:** 10.3390/vaccines14070581

**Published:** 2026-06-30

**Authors:** Ren Yang, Xiaohong Wu, Xiaowei Zhang, Shengqing Fu, Kaiping Gu, Zhe Lv, Xiaoli Li, Qunying Mao

**Affiliations:** 1 National Institutes for Food and Drug Control, Beijing 102629, China; yangren@nifdc.org.cn (R.Y.);; 2 State Key Laboratory of Drug Regulatory Science, Beijing 100050, China; 3 Tianjin Pharmaceutical and Cosmetic Evaluation and Inspection Center, Tianjin 300308, China; 4 Department of Analytical Science, Suzhou Abogen Biosciences Co., Ltd., Suzhou 215123, China; 5 Sinovac Life Sciences Co., Ltd., Beijing 102601, China; 6 Shandong WeigaoLitong Biological Products Co., Ltd., Weihai 276300, China

**Keywords:** mRNA vaccine, quality control, capping efficiency, LC-MS

## Abstract

**Background:** The m^7^G cap structure, which mimics the natural cap of eukaryotic mRNA, is a critical determinant of mRNA vaccine efficacy, safety, and stability. However, its precise quantification remains challenging due to complex impurity profiles and the high physicochemical similarity between the target cap and related impurities. Although liquid chromatography mass spectrometry (LC-MS) is widely employed for this purpose, current methodologies still face significant limitations, including labor-intensive sample preparation, low analytical throughput, poor reproducibility in quantifying low-level impurities, and a lack of universally applicable strategies across diverse mRNA vaccine platforms. **Methods**: We systematically optimized sample preparation and LC-MS detection workflows. RNase H-mediated cleavage was compared with DNAzymes, guide DNA probes were rationally designed, and thermostable RNase H was introduced for one-step denaturation and cleavage. To establish an accurate, efficient, and universal sample preparation workflow. Chromatographic conditions were optimized using an ion-pairing reagent system to suppress ESI-MS metal adducts. Eliminating sample purification improves recovery, reduces manual handling errors, and boosts assay efficiency. **Results**: Through optimally designed guide DNA probes, RNase H cleavage specificity reached ≥98% with high cleavage efficiency, offering higher efficiency than DNAzyme. Furthermore, the incorporation of thermostable RNase H enabled a single-step workflow combining high-temperature denaturation and site-specific cleavage, substantially streamlining sample preparation. On the chromatographic side, optimization of the ion-pairing reagent system effectively suppressed metal adduct formation in electrospray ionization mass spectrometry (ESI-MS). This advancement enabled direct injection of the 5′ cap fragments without purification, achieving high-recovery quantification while demonstrating broad compatibility across mainstream LC-MS platforms. The optimized assay reduces the total analytical workflow from 4~6 h to under 1.5 h. **Conclusions:** Combining high accuracy, robustness, and broad platform compatibility, this method offers a universal, high-throughput analytical solution for mRNA vaccine quality control and continuous process development.

## 1. Introduction

The 5′ cap structure, featuring a 7-methylguanosine (m^7^G) residue linked via a 5′-5′ triphosphate bridge, is essential for eukaryotic mRNA stability, translational competence, and immune evasion [1,2]. The physiological Cap1 structure (m^7^GpppNm), characterized by 2′-O-methylation of the first transcribed nucleotide, enables cellular innate immune sensors to distinguish self-RNA from exogenous transcripts, thereby preventing unintended immune activation [3,4]. Given its critical role, precise mimicry or incorporation of an intact cap structure is a cornerstone technology in the manufacturing of synthetic mRNA vaccines and therapeutics, directly governing transcript stability and translational yield [5,6,7,8].

Modern mRNA production primarily employs enzymatic or co-transcriptional capping. Enzymatic cascades sequentially add m^7^G and 2′-O-methyl groups but frequently generate structural impurities, including uncapped RNA (pppN), unmethylated G-caps (GpppN), Cap0 structures (m^7^GpppN), and degradation products (pppNm) [2,9,10,11,12]. Co-transcriptional capping using cap analogs, particularly the CleanCap^®^ series (e.g., m^7^GpppAmpN and 3′-O-Me-m^7^GpppAmpN), leverages T7 RNA polymerase initiation preference to improve efficiency and has become the mainstream approach for bulk production [13,14,15,16,17]. Because co-transcriptional capping circumvents the formation of intermediates such as GpppN and Cap0 at the raw material level, its impurity profile is relatively simplified, with the primary theoretical residuals being unincorporated pppAN-initiated RNAs and cap degradation products (pppAmN) [9]. The bioactive Cap1 species in mRNA vaccines exhibits minimal physicochemical differences from other impurities, and impurity levels are typically low. Consequently, highly precise analytical methods are required for accurate quantification.

Accurate determination of capping efficiency relies on two core steps: the specific release of the mRNA 5′ terminal fragment and subsequent instrumental quantification [18,19]. The key to sample preparation lies in the efficient separation of the 5′ cap region from the full-length mRNA backbone. Current mainstream strategies include RNase H-mediated cleavage and DNAzyme-based cleavage. RNase H utilizes chimeric DNA-RNA probes for site-specific cleavage but requires careful sequence design to ensure specificity [9,19]. Conversely, ribozyme strategies offer high site specificity but are constrained by strict sequence motif requirements within mRNA untranslated regions (UTRs) and often exhibit lower substrate conversion efficiency [20,21]. To date, comprehensive comparative evaluations and systematic optimization of sample preparation strategies remain underexplored in the literature.

Common instrumental techniques for capping efficiency assessment include high-performance liquid chromatography (HPLC), capillary electrophoresis (CE), and liquid chromatography–mass spectrometry (LC–MS), with HPLC and LC–MS/MS now incorporated into pharmacopeial guidelines such as the USP [22,23]. HPLC, CE, and electrophoresis separation behavior are heavily dependent on nucleotide sequence and fragment length; consequently, retention times, resolution, and limits of detection fluctuate with target sequences, limiting method universality [24,25]. In contrast, LC–MS does not require complete chromatographic separation of capped and uncapped species, as quantification can be achieved based on m/z differences, offering superior sequence independence [9,21]. However, oligonucleotide fragments readily form complex metal adduct peaks during electrospray ionization due to interactions with mobile phase or matrix ions (e.g., Na^+^, K^+^, Ni^2+^), complicating spectral interpretation [26,27]. Consequently, conventional LC–MS workflows mandate time-consuming desalting or purification steps to remove digestion buffer salts, increasing procedural complexity and analysis time.

To address these bottlenecks, this study systematically optimizes both sample preparation and LC–MS analysis to develop a rapid, robust, and universal capping efficiency assay. We first constructed mRNA reference materials containing randomized sequences using both enzymatic and co-transcriptional capping processes to comprehensively cover typical capped and uncapped impurity profiles. We then evaluated RNase H versus DNAzyme cleavage strategies to establish an optimal digestion protocol. Crucially, by optimizing ion-pairing reagents in the mobile phase, we effectively suppressed metal adduct formation during mass spectrometric detection. This advancement eliminates the need for traditional purification steps, enabling direct injection of cleavage products and reducing the total workflow from 4–6 h to under 1.5 h. The optimized assay demonstrated favorable recovery and precision across diverse 5′ terminal fragments while maintaining broad compatibility with mainstream LC–MS platforms. Validation across multiple batches of mRNA vaccine bulk materials from various manufacturers confirmed its practical utility for industrial quality control and batch release testing, providing a high-throughput analytical tool for accurate capping efficiency evaluation in mRNA therapeutics.

## 2. Materials and Methods

### 2.1. Sequence Design and Probe Synthesis

The in vitro transcription (IVT) mRNA template constructed in this study comprises a human IgG1 5′ untranslated region (UTR), a 1000-nt randomized non-coding region (serving as a coding sequence length surrogate), and a human ACTN3 3′ UTR. Specific nucleotides within the 5′ UTR were site-directed mutagenized to accommodate subsequent cleavage site studies. The DNA template was chemically synthesized by GenScript (Nanjing, China). The DNAzyme probe featured a 10–23 catalytic core flanked by two 15-nt hybridization arms fully complementary to the target mRNA sequence. The RNase H cleavage probe was designed as a DNA–RNA chimera: the non-cleavage region utilized a 3′-O-methyl RNA backbone, while the RNase H recognition site consisted of a DNA sequence. A biotin–TEG linker was conjugated to the 3′ terminus. The theoretical melting temperature (*T_m_*) of the probes was calculated to be 55–65 °C using OligoCalc. Both the DNAzyme and RNase H probes were synthesized by GenScript, with purity verified by HPLC and mass spectrometry (>95%), and used as received.

### 2.2. In Vitro Transcription and mRNA Capping

The DNA template containing a T7 promoter was linearized using *BspQ* I restriction endonuclease (New England Biolabs, NEB, Ipswich, MA). Transcription was performed according to the manufacturer’s instructions for the T7 IVT kit (Novoprotein, Shanghai, China). The reaction product was purified via LiCl precipitation to yield uncapped mRNA. Subsequently, Cap1 structures (m^7^GpppGm*) were added using a vaccinia virus capping system (Novoprotein), followed by a second LiCl precipitation. The integrity and purity of the purified mRNA were assessed using an Agilent 5300 Bioanalyzer (Agilent Technologies, Santa Clara, CA, USA) (RNA High Sensitivity chip). Concentration was determined dually using a NanoDrop 2000 spectrophotometer (Thermo Fisher Scientific, Waltham, MA, USA) (*A*_260_) and a Qubit 4.0 fluorometer (Thermo Fisher Scientific, Waltham, MA, USA) (RiboGreen assay).

### 2.3. DNAzyme Cleavage Reaction

mRNA and DNAzyme were mixed at a 1:4 molar ratio, denatured at 85 °C for 5 min, and annealed to room temperature at a ramp rate of 0.2 °C/s. Cleavage buffer of varying pHs was added to a final concentration of 10 mM Tris–HCl, 50 mM NaCl, and 20 mM magnesium acetate diluted with RNase-free water (Invitrogen, Carlsbad, CA, USA). Each 100 μL reaction contained 100 pmol of mRNA (1 μM). A total of 5 units of calf intestinal alkaline phosphatase (CIAP, Takara, Otsu, Japan) was added to each reaction. Reactions were incubated at 37 °C for 15, 30, 60, 120, and 180 min, then terminated by adding 1/10 volume of 0.2 M EDTA·NH_4_OH. Products were purified using the Monarch^®^ Spin RNA Cleanup Kit (NEB) prior to LC–MS analysis.

### 2.4. RNase H Cleavage and Specificity Analysis

For *E. coli* RNase H (NEB) cleavage, mRNA and probe were mixed at a 1:2 molar ratio, denatured at 95 °C for 3 min, and annealed at 0.1 °C/s to room temperature. Ten units of RNase H were added per 100 pmol of RNA in a final volume of 100 μL. The *Thermus thermophilus* thermostable RNase H (NEB) system followed identical conditions, with the incubation temperature set to 50 °C. Reactions were terminated with a 1/10 volume of 0.2 M EDTA·NH_4_OH. For cleavage efficiency evaluation, reactions were incubated for 15, 30, 60, 120, and 180 min; a 30 min incubation was standardized for all subsequent method development and sample testing. *E. coli* RNase H digestion products were captured and purified using streptavidin magnetic beads (Invitrogen). To assess cleavage specificity, a chemically synthesized 50 nt RNA oligonucleotide (corresponding to the mRNA 5′-terminal sequence, GenScript) was used in place of full-length mRNA under identical conditions. The specificity index was calculated by quantifying the relative abundance of the target cleavage fragment versus non-specific byproducts via LC–MS.

### 2.5. Cleavage Efficiency Detection

Residual intact mRNA was quantified using a one-step reverse transcription droplet digital PCR (RT-ddPCR). Primers were designed to span the cleavage site, with a TaqMan probe positioned approximately 50 nt from the 5′ end (5′-FAM, 3′-BHQ1). The One-Step RT-ddPCR™ Advanced Kit for Probes (Bio-Rad, Hercules, CA, USA) was used. Each 20 μL reaction contained 2 μL of 1:4000 diluted cleavage product or control mRNA, with final primer and probe concentrations of 900 nM and 250 nM, respectively. After droplet generation, reverse transcription was performed at 50 °C for 1 h. The PCR program consisted of initial denaturation at 95 °C for 10 min, followed by 40 cycles of 95 °C for 30 s and 58 °C for 1 min, and enzyme deactivation at 98 °C for 10 min. Fluorescence signals were acquired using a QX200 Droplet Reader (Bio-Rad) to calculate absolute copy numbers. Cleavage efficiency was calculated as follows:

(1) Cleavage efficiency (%) = [1 − (*N_treated_*/*N_control_*)] × 100%

where *N_treated_* and *N_control_* represent the copies/μL numbers of intact mRNA in the treated and untreated control groups, respectively.

### 2.6. Chromatographic Conditions

For LC–MS analysis, organic solvents included triethylamine (TEA, >99%; Sigma-Aldrich, St. Louis, MO, USA), (DIPEA, >99.5%; Aladdin, Shanghai, China), hexafluoroisopropanol (HFIP, >99.5%; Aladdin, Shanghai, China), diisopropylethylamine, and LC–MS grade methanol (Honeywell, Charlotte, NC, USA), used as received.

Mobile phases were prepared as follows:

Aqueous phase A: 8 mM TEA and 200 mM HFIP;

Aqueous phase B: 6 mM DIPEA and 200 mM HFIP;

Aqueous phase C: 12.5 mM DIPEA and 25 mM HFIP;

Organic phase: pure methanol.

All mobile phases were degassed by ultrasonication for 15 min. Chromatographic separation was performed on an Agilent 1290 Infinity II UPLC system equipped with a DNAPac RP 4 μm, 2.1 × 100 mm column (Thermo Scientific, Waltham, MA, USA), maintained at 60 °C. The gradient program (flow rate: 0.3 mL/min) was as follows: 0–5 min, 5% organic phase; 5–15 min, 5% to 35% organic phase; 15–16 min, 35% to 90% organic phase; 16–18 min, 90% organic phase; 18–20 min, return to 5% organic phase. The elution window for target cap fragments was 5–11 min, with the effluent directed directly to the mass spectrometer.

### 2.7. Mass Spectrometry Detection and Data Processing

Samples (10 μL injection, corresponding to 10 pmol of total mRNA) were analyzed on an Agilent 6550 iFunnel Q-TOF mass spectrometer (Agilent Technologies) equipped with a Dual AJS ESI source operating in negative ion mode. Source parameters were as follows: desolvation gas temperature 275 °C/flow 12 L/min; sheath gas temperature 350 °C/flow 10 L/min; capillary voltage 3.5 kV; nozzle voltage 1.0 kV. Full-scan data were acquired in the m/z range of 600–3000. Raw data were processed using Agilent BioConfirm 12 software. Charge-state deconvolution was performed using the maximum entropy algorithm, filtering for ion clusters comprising ≥3 consecutive charge states within a mass tolerance of ±150 ppm. Target analyte abundance was quantified by summing the peak areas of all deconvoluted target species.

Regarding cross-platform applicability of the optimized method, mRNA bulk samples from three different manufacturers were analyzed on corresponding mass spectrometry platforms: Manufacturer A: Agilent 6545 Q-TOF mass spectrometer (Agilent Technologies, Santa Clara, CA, USA) with data processed using the maximum entropy deconvolution algorithm in BioConfirm 10 software; Manufacturer B: Waters G2-XS Q-TOF mass spectrometer (Waters Corporation, Milford, MA, USA) with data processed using the BayesSpray deconvolution algorithm in intact-mass software; Manufacturer C: Thermo Q Exactive Plus Orbitrap mass spectrometer (Thermo Fisher Scientific, Waltham, MA, USA) with data processed using the Xtract deconvolution algorithm in BioPharma Finder 5.0 software.

### 2.8. Statistical Analysis

Raw data were organized using WPS Office. Statistical analysis and data visualization were performed with GraphPad Prism 9.0. Recovery rates were evaluated via linear regression analysis. Comparisons among multiple groups were conducted using one-way analysis of variance (ANOVA) followed by Tukey’s multiple comparisons test. A *p*-value of <0.05 was considered statistically significant.Statistical significance is indicated as ns *p* > 0.05, * *p* < 0.05, ** *p* < 0.01 and *** *p* < 0.001.

## 3. Results

### 3.1. Deoxyribozyme Cleavage Efficiency Is Influenced by Reaction pH and Target Sequence Motif

Deoxyribozymes are catalytic DNA molecules characterized by specific secondary and tertiary structures, enabling sequence-specific recognition and site-directed cleavage of target RNA strands. Their advantages include high compatibility with diverse reaction systems, well-defined metal ion dependence, and highly specific cleavage sites. Currently, deoxyribozyme probes widely employed for site-specific RNA cleavage are primarily classified into the 8–17 and 10–23 families [28,29]. The 8–17 DNAzyme strictly requires an “AG” dinucleotide motif, resulting in a rigid cleavage site that is difficult to adapt to the varying 5′ untranslated region (UTR) sequences of different mRNAs [30]. In contrast, the 10–23 DNAzyme recognizes and cleaves adjacent purine–pyrimidine (R↓Y) base pairs, offering greater flexibility in cleavage site selection [31]. Given these characteristics, this study focuses on the application potential of the 10–23 DNAzyme probe (incorporating a DNA guide sequence to enhance synthetic stability) for releasing the mRNA 5′-terminal fragment.

**Figure 1:**
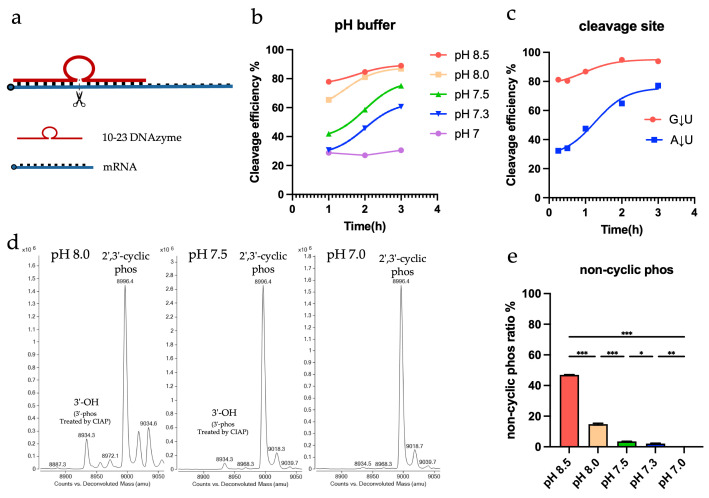
DNAzyme cleavage efficiency and analysis of cleavage products. (**a**) Schematic illustration of the DNAzyme-mediated cleavage mechanism. (**b**) Cleavage efficiency of the 10–23 DNAzyme at the G↓U site across a range of pH conditions. (**c**) Comparative cleavage efficiency of the 10–23 DNAzyme at two distinct target sites at pH 8.5. (**d**) Deconvoluted mass spectra of cleavage products under different pH conditions. (**e**) Relative proportions of 3′-terminal free phosphate fragments generated across varying pH levels. * *p* < 0.05, ** *p* < 0.01 and *** *p* < 0.001.

Compared to protein nucleases, ribozymes and DNAzymes typically exhibit lower catalytic turnover rates (*k_cat_*). To overcome this efficiency bottleneck, we investigated the impact of reaction pH on the catalytic activity of the 10–23 DNAzyme. The results demonstrated that elevated pH enhanced DNAzyme-mediated cleavage efficiency. (Figure 1b). Site preference analysis revealed that cleavage at the G↓U dinucleotides proceeded at a significantly higher rate than at the A↓U sites, which is consistent with previous literature reports [31]. Although reaction kinetics could be enhanced through condition optimization, the maximum cleavage conversion of mRNA remained approximately 90%, even after a 3 h incubation (Figure 1c).

Upon LC–MS analysis of the aforementioned cleavage products, we observed that while high pH conditions effectively promoted RNA backbone cleavage, they simultaneously altered the terminal chemical structure of the cleavage products. Under alkaline conditions, the 2′,3′-cyclic phosphate intermediate normally generated by DNAzyme activity undergoes non-specific hydrolysis, extensively converting into a 3′-phosphate terminus (confirmed in this study following treatment with alkaline phosphatase). These chemically modified byproducts not only shifted the chromatographic retention behavior of the target oligonucleotide fragments but also caused broadening of isotopic envelopes and signal dispersion during mass spectrometric detection. Additionally, a column chromatography purification step was included to mitigate potential mass spectrometric interference from relatively high Mg^2+^ concentrations. Consequently, this compromised the accurate quantification and spectral interpretation of the target fragments (Figure 1d,e).

### 3.2. Sequence-Dependent Analysis of Cleavage Specificity and Efficiency of RNase H Cleavage

**Figure 2:**
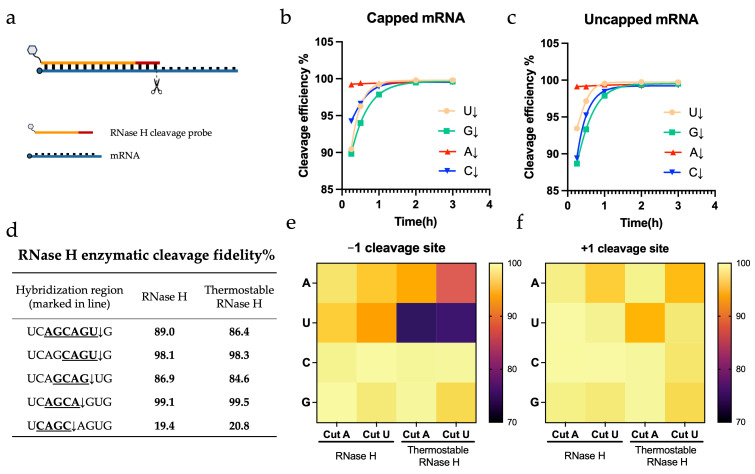
RNase H cleavage specificity and efficiency. (**a**) Schematic illustration of the RNase H-mediated cleavage mechanism. (**b**,**c**) Cleavage efficiency for capped and uncapped mRNA when targeting different nucleotide bases. (**d**) Impact of hybridization probe design on cleavage specificity. (**e**,**f**) Effect of flanking nucleotide sequences at the +1 and −1 positions relative to the cleavage site on RNase H cleavage specificity when targeting adenine (A) or uracil (U) residues.

Ribonuclease H (RNase H) is a family of endonucleases that specifically hydrolyze the RNA strand within DNA–RNA hybrid duplexes. Its catalytic activity exhibits low sequence dependence and high cleavage efficiency [9,24]. Leveraging this property, the design of chimeric probes complementary to the 5′ terminus of mRNA enables precise release of 5′-terminal oligonucleotide fragments containing the cap structure, providing a robust sample preparation protocol for subsequent LC–MS quantification of capping efficiency [32,33]. To balance cleavage efficiency with site specificity, we systematically evaluated the impact of hybridization region length on RNase H cleavage behavior. A comparison of probes with 6-nt versus 4-nt DNA–RNA hybridization regions revealed that both achieved high substrate conversion; however, the 6-nt region was prone to off-target cleavage, whereas the 4-nt region significantly enhanced cleavage specificity by 9–12% (Figure 2d).

Using the 4-nt hybridization probe, we further compared the sequence preferences of *E. coli* RNase H and *Thermus thermophilus* thermostable RNase H. The results indicated that when the 3′-terminal nucleotide of the RNA strand within the hybrid duplex (i.e., the cleavage site) was adenine (A) or uracil (U), both enzymes exhibited optimal cleavage specificity (≥98%, Figure 2d). Although the overall sequence dependence of the two enzymes was comparable, *E. coli* RNase H demonstrated higher site fidelity in certain sequence contexts. Notably, when the nucleotide at the −1 position upstream of the cleavage site was U, specificity decreased for both enzymes, with the thermostable RNase H showing greater sensitivity to this motif (Figure 2e,f). Furthermore, the combination of the cleavage site and the downstream +1 nucleotide also influenced cleavage precision. We therefore recommend avoiding A↓U or U↓A motif in practical probe design.

In kinetic evaluations, RNase H exhibited maximal catalytic efficiency when the 3′-terminal nucleotide of the hybrid RNA was A. Under optimized conditions (100 pmol mRNA, 10 U RNase H), >99% substrate conversion was achieved within 15 min (Figure 2b,c). This high-efficiency cleavage showed no significant difference between capped (Cap0/Cap1) and uncapped (pppN) mRNA molecules, ensuring matrix consistency for quantitative analysis. In summary, the systematically optimized RNase H probe design strategy simultaneously meets the dual requirements of high specificity and high efficiency. Consequently, this sample preparation protocol was adopted for the assessment of mRNA capping efficiency in subsequent analyses. Subsequently, our focus shifted to optimizing mass spectrometric detection conditions to enhance instrumental analytical efficiency.

### 3.3. Optimization of Ion-Pair Reversed-Phase Chromatography Mobile Phase to Suppress Metal Adduct Interference

Ion-pair reversed-phase chromatography (IP-RPC) is a highly efficient technique for separating nucleic acid fragments of varying lengths. Conventional systems typically employ organic amine-based ion-pairing reagents, such as triethylamine, diisopropylethylamine, or dibutylamine, often coupled with acetic acid to adjust pH and ionic strength [34,35,36]. Previous studies have demonstrated that substituting acetic acid with hexafluoroisopropanol as a mobile phase additive provides a stable proton source and optimizes the desolvation process of electrospray ionization (ESI) droplets, thereby significantly enhancing the mass spectrometric response of nucleic acids in negative ion mode [37]. Consequently, HFIP-based systems have emerged as the preferred choice for oligonucleotide LC–MS analysis [38].

However, the phosphate backbone of nucleic acids exhibits a strong affinity for metal cations, readily forming complex metal adduct peaks (e.g., Na^+^, K^+^, Ni^2+^) during ESI–MS [39]. For chemically synthesized oligonucleotides, adduct formation can be mitigated by dissolving samples in metal-free solvents and strictly controlling the metal background of the mobile phase. In contrast, the determination of mRNA capping efficiency relies on metal-ion-dependent enzymatic cleavage to release the 5′-terminal fragments, inevitably introducing metal cofactors into the reaction matrix. Even after purification steps such as solid-phase extraction or magnetic bead cleanup, residual metal ions can still cause significant adduct interference, compromising the abundance and quantitative accuracy of the target deprotonated ions ([M − nH]^n−^).

**Figure 3:**
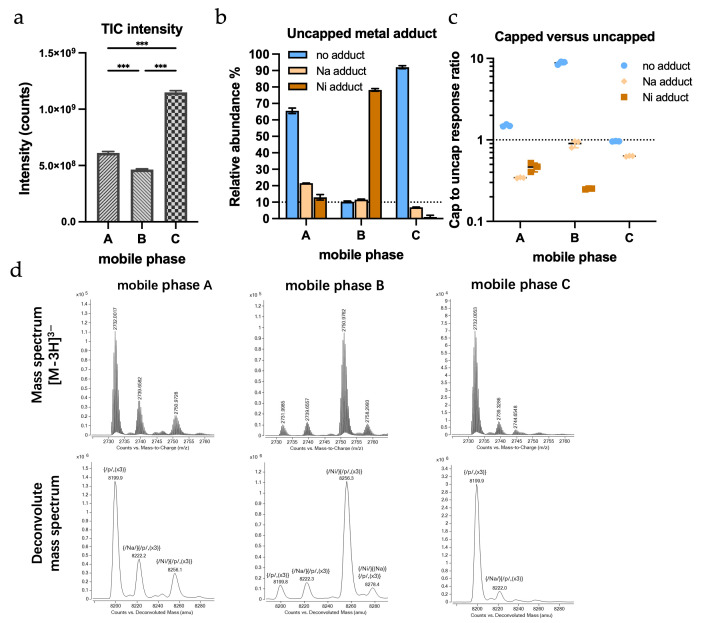
Metal adduct profiles of nucleic acid fragments under mobile phases with different ion-pairing reagent combinations. Mobile phase compositions: *A* 8 mM TEA + 200 mM HFIP in water; *B* 6 mM DIPEA + 200 mM HFIP in water; *C* 12.5 mM DIPEA + 25 mM HFIP in water. The organic phase consisted of pure methanol. (**a**) TIC peak response intensities of the target fragments. (**b**) Relative proportions of metal ion adducts for uncapped fragments across different mobile phases. (**c**) Ratios of adduct ion responses between capped and uncapped mRNA in mass spectrometric analysis. (**d**) Raw mass spectra and deconvoluted mass spectral data of uncapped components from unpurified enzymatic cleavage products, analyzed under different mobile phase conditions. *** *p* < 0.001.

To overcome this bottleneck, we systematically optimized the ion-pairing chromatographic system (mobile phase C in Figure 3). Compared to conventional formulations, increasing the concentration of the cationic ion-pairing reagent DIPEA to 12.5 mM while reducing HFIP to 25 mM effectively modulated the binding equilibrium between phosphate groups and metal ions. This adjustment significantly suppressed metal adduct formation (Figure 3d) and concurrently enhanced the signal response of target analytes (Figure 3a). Under these optimized conditions, enzymatic mRNA digestion products could be directly injected without any prior purification. The results demonstrated that mono-metallic adducts (e.g., Na^+^, K^+^) were suppressed to <10% of the primary deprotonated ion ([M − H]^−^) response (Figure 3b,d), while multivalent metal adducts (e.g., Ni^2+^) were virtually eliminated. Crucially, this condition not only substantially boosted the MS response of target RNA fragments but also equalized the metal adduct distribution profiles between capped (Cap0/Cap1) and uncapped (pppN) species (Figure 3c). This response equilibrium mitigates ionization efficiency biases arising from differential adduct ratios, thereby enhancing the accuracy and robustness of subsequent target ion extraction, charge-state deconvolution, peak area integration, and capping efficiency calculations. Therefore, this optimized mobile phase composition enables direct injection analysis of high-salt samples without prior purification.

### 3.4. Establishment of a Purification-Free Rapid LC–MS Assay for mRNA Capping Efficiency Using Thermostable RNase H and Metal Adduct Suppressed Mobile Phase

*E. coli* RNase H typically catalyzes reactions at 37 °C, requiring prior high-temperature denaturation to resolve mRNA secondary structures. However, the relatively low reaction temperature often induces probe slippage and non-specific cleavage, generating extensive nucleic acid debris. Without purification, significant impurity interference commonly obscures the target chromatographic peaks, necessitating desalting or magnetic bead cleanup steps. In contrast, thermostable RNase H enables direct enzymatic cleavage at elevated temperatures. This high-temperature environment not only eliminates the pre-denaturation step but also significantly enhances the hybridization specificity of the DNA probe to the target sequence, thereby fixing the cleavage site. This characteristic substantially improves chromatographic peak purity, laying the foundation for direct injection analysis. Coupled with the aforementioned low metal adduct mobile phase system, we successfully established a rapid, purification-free LC–MS method for quantifying mRNA capping efficiency.

To address the issue of heterogeneous phosphorylation states in uncapped RNA or cap degradation products—where 5′-phosphate groups are prone to non-enzymatic hydrolysis—we comparatively evaluated the inclusion of CIAP in the digestion buffer. CIAP specifically hydrolyzes exposed 5′-phosphate esters, uniformly converting various degraded/uncapped fragments to 5′-hydroxyl termini, thereby normalizing the mass and charge states of target ion peaks. Mass spectrometric data were processed using a maximum entropy isotopic deconvolution algorithm. Raw MS data within the target chromatographic peak were extracted, with a mass tolerance window of ±150 ppm. The relative abundances of the primary deprotonated ions ([M − nH]^n−^) and their mono-alkali metal adducts (e.g., [M + Na − nH]^n−^, [M + K − nH]^n−^) were cumulatively calculated to mitigate the impact of adduct signal dispersion on quantitative accuracy.

**Figure 4:**
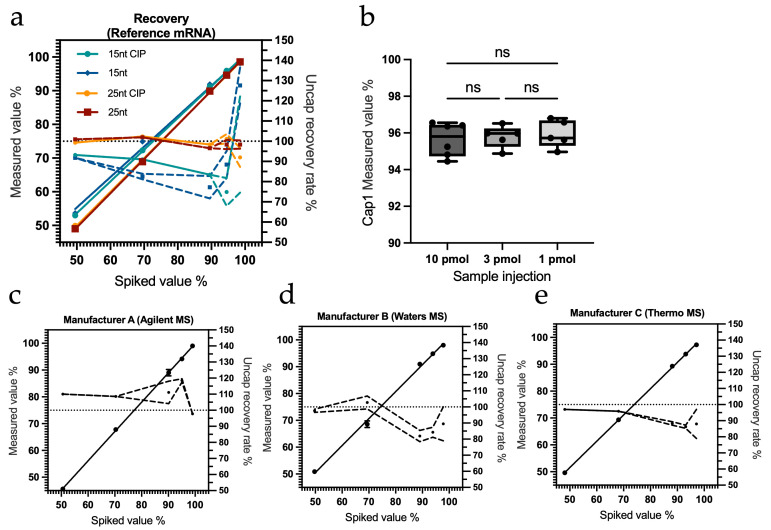
Spike recovery validation and precision at low injection volumes for capped and uncapped species. (**a**) Spike recovery validation using reference mRNA. (**b**) Quantification results and precision at injection volumes ranging from 1 to 10 pmol. (**c**–**e**) Spike recovery validation for capping efficiency in mRNA bulk material from manufacturers A, B, and C. Dashed lines indicate the SD range.

Method validation was conducted across a spiking range corresponding to an mRNA capping value of 49.54–98.54%, achieved by spiking at 1%, 5%, 10%, 30%, and 50% (*w*/*w*) uncapped impurity into a high-purity bulk material (99.54% capped). Table 1 presents the recovery data. Column headers indicate the theoretical Cap or Uncap value. For each cleavage fragment and preparation, the table lists the measured Uncap values, RSD (*n* = 3), and the mean recovery (%). The results demonstrated that this strategy yielded satisfactory recovery for 5′-terminal cleavage fragments of varying lengths (15 nt and 25 nt), with good precision across all uncapped spiking levels (RSD < 10%, *n* = 3), except at the 98.54% recovery point for the 15 nt CIAP-treated sample, where the RSD exceeded 20% (Table 1, Figure 4a). Notably, the detection recovery for 25 nt fragments was significantly superior to that of 15 nt fragments (*p* < 0.01), likely attributable to more stable retention in reversed-phase chromatography and higher ESI efficiency for longer oligonucleotides (Table 1). The addition of CIAP slightly improved the linearity of recovery for 15 nt fragments (*R*^2^ increased from 0.996 to 0.999) but had a limited overall impact on recovery. Consequently, the 25 nt digestion protocol without CIAP was selected for subsequent methodological investigations. By eliminating complex procedures such as high-temperature reannealing and magnetic bead purification, and reducing instability factors from metal adducts in data analysis, this method exhibited ideal intra-day and inter-day precision (Table 2). It performed robustly for samples with conventional capping levels (95 ± 1%) and those approaching the quality specification threshold (~90%). Furthermore, we evaluated the sensitivity and low-level detection capability by reducing the standard injection amount of a Cap1 mRNA sample (prepared via enzymatic capping) from 10 pmol to 3 pmol and 1 pmol. Analysis indicated that uncapped components remained detectable even at 1 pmol, with no significant differences across loading concentrations (Figure 4b); however, the proportions of low-abundance impurity components (e.g., pN) exhibited moderate fluctuations.

To further assess cross-platform versatility, the method was applied to the analysis of in-development mRNA bulk substances from three different manufacturers employing distinct capping processes. Despite variations in mass spectrometry platform differences in ion source designs affecting nucleic acid ionization efficiency, and divergent deconvolution algorithm parameters in vendor software, the method demonstrated consistent recovery performance across all platforms (Figure 4c–e). These findings indicate that the optimized protocol can effectively overcome hardware and software algorithmic discrepancies, exhibiting desired adaptability and robustness across diverse mRNA products and mainstream LC–MS systems. This validates its suitability for industrial quality control and lot release testing.

### 3.5. Capping Efficiency Assay of mRNA Vaccine Bulk Substances Across Multiple Manufacturers

Subsequently, we applied this rapid LC–MS quantification method to evaluate the capping efficiency of multiple in-development mRNA bulk substances and systematically characterized their major impurity profiles (Table 3).

The majority of the tested candidates utilized co-transcriptional capping. Notably, we found that a fully optimized enzymatic capping process could also achieve exceptionally high capping efficiency, with uncapped species present only at trace levels. For co-transcriptionally capped products, capping efficiencies consistently exceeded 94%. Interestingly, Cap0-like impurities (e.g., m^7^GpppN or GpppNm) were still detectable in certain co-transcriptional batches. This observation suggests potential batch-to-batch variability in the *in-vitro* transcription (IVT) raw materials, highlighting the need for enhanced quality control of critical starting materials. Furthermore, structural characterization revealed that uncapped fragments derived from co-transcriptional capping typically retained an intact 5′-triphosphate (5′-pppN) moiety, whereas cap degradation products predominantly exhibited a 5′-monophosphate (5′-pN) terminus.

## 4. Discussion

The Cap1 structure is a critical determinant of efficient translation, evasion of innate immune recognition, and in vivo stability of mRNA vaccines in eukaryotic cells [1,4,5]. Consequently, capping efficiency directly correlates with vaccine immunogenicity, safety, and batch-to-batch consistency. However, in full-length mRNA molecules (typically 1000–4000 nt), the structural differences between Cap1-modified transcripts and uncapped or degraded impurities are confined to the 5′ end, making precise discrimination challenging using conventional intact molecule-based assays. Therefore, the specific release of the 5′ cap region to amplify its physicochemical differences is a prerequisite for accurate quantification [9,19,21]. In this study, we compared two site-specific cleavage strategies: DNAzymes and RNase H. By optimizing the hybridization domain of the guide probe for RNase H digestion, the cleavage site was restricted to adenine (A) or uracil (U) nucleotides, with a preference for A sites, showing great enzymatic fidelity. Especially, the adenine cleavage site approach achieved >99% substrate conversion within 15 min, with markedly improved site specificity. Although RNase H exhibits inherent sequence bias, which somewhat constrains the flexibility in selecting high-fidelity cleavage sites, it still offers considerably greater tolerance in site selection compared to DNAzyme systems. This highly efficient, high-fidelity, and broadly applicable sample preparation strategy not only maximizes the signal response of the target fragment but also effectively eliminates interference from non-specific cleavage products in both chromatographic separation and mass spectrometric quantification windows, thereby establishing a high-purity matrix for downstream instrumental analysis.

Traditional capping efficiency assays heavily rely on post-cleavage purification steps using biotin-streptomycin magnetic beads or solid-phase extraction columns, which are time-consuming (typically 4–6 h) and prone to sample loss and inter-batch variability [9,20]. In this work, we innovatively combined the high-temperature cleavage capability of thermostable RNase H with an ion-pair reversed-phase chromatography (IP-RPC) mobile phase system designed to minimize metal adduct interference. This approach successfully enabled direct injection analysis without purification, reducing the total workflow time to under 1.5 h. Mechanistic investigations revealed that the synergistic optimization of the cationic ion-pairing reagent concentration and the absolute content of HFIP in the mobile phase is crucial for suppressing metal adducts [38]. High concentrations of protonated amines competitively bind to coordination sites on the nucleic acid phosphate backbone, effectively displacing alkali metal ions such as Na^+^ and K^+^. Concurrently, reducing the HFIP concentration lowers the overall proton/cation load in the system, further mitigating the formation of multiply charged adducts [34,36,39]. Additionally, the cleavage reaction termination step incorporated EDTA dissolved in ammonium hydroxide, which chelates residual metal cofactors, thereby suppressing the overall metal adduct background to minimal levels. This strategy ensures high chromatographic peak purity while completely eliminating laborious purification steps, significantly enhancing analytical throughput and method robustness.

Oligonucleotides readily form multiply charged ion clusters in electrospray ionization mass spectrometry (ESI-MS), and variations in ion source design and ionization parameters across different mass spectrometer manufacturers can cause fluctuations in mass-to-charge ratio (*m*/*z*) distributions. To overcome hardware dependency, we employed a maximum entropy deconvolution algorithm based on average molecular weight for data processing. By setting a mass tolerance window of ±150 ppm, the algorithm accurately matched target species and summed the abundances of their singly metal adducted ions. Although deconvolution models in vendor-specific software differ algorithmically, method validation demonstrated that this strategy yields acceptable consistent quantitative accuracy across a capping rate range of 50% to 99% (recovery for impurities ranged from 80% to 120%, with RSD < 10%). This confirms the robustness of our method to variations in instrumental hardware and software algorithms, enabling seamless adaptation to diverse LC-MS platforms across laboratories and meeting analytical demands ranging from early process development to commercial release testing.

Although the United States Pharmacopeia (USP) guidelines recommend high-performance liquid chromatography (HPLC) for capping rate determination, and several manufacturers have established corresponding methods, practical widespread application faces notable limitations [23,24]. HPLC separation behavior is highly dependent on the nucleic acid sequence, fragment length, 5′ untranslated region (5′ UTR) design, column stationary phase, and mobile phase gradient, necessitating method redevelopment for each distinct mRNA candidate. Furthermore, peak identification relies heavily on high-purity uncapped reference standards, which are particularly challenging to synthesize via co-transcriptional capping [17]. While biological assays for capping efficiency exist, they lack precise quantification of individual impurity species, limiting their utility in product process optimization [40,41,42,43]. In contrast, the LC-MS strategy does not require complete chromatographic resolution; instead, it directly quantifies characteristic ions based on m/z differences. This approach exhibits strong tolerance to sequence variations, capping methodologies, and fragment lengths, while enabling accurate quantification of all impurity components. Consequently, it provides an efficient and universal solution for the quality control of mRNA products.

The rapid capping rate assay developed in this study demonstrated satisfactory recovery and detection performance for mRNA samples with capping rates between 50% and 99%. However, due to technical constraints, we were unable to obtain a 100% Cap1-capped reference material; the highest capping efficiency achieved for our in-house reference standard was 99.54%. To simulate the detection limit for trace impurities, we evaluated reduced injection volumes (1/10 of the standard load), which still enabled accurate identification and quantification of impurity components. This suggests that the method can achieve a desirable limit of detection (LOD) for ultra-low-abundance impurities. Nevertheless, a more comprehensive LOD assessment still requires further systematic validation.

We further applied this method to analyze in-process mRNA bulk materials from multiple manufacturers utilizing different capping strategies (enzymatic and co-transcriptional). The results indicated that the Cap1 ratio remained consistently high across all batches, with ideal inter-batch precision. Notably, samples approaching their expiration date maintained exceptional capping stability under specified frozen storage conditions, with only trace amounts of cap degradation products detected, fully complying with predefined quality specifications. This underscores the protective efficacy of current mainstream manufacturing processes and storage conditions on the cap structure. Interestingly, while most mRNA bulks produced via co-transcriptional capping with cap analogs showed no detectable Cap0 or G-cap species, a subset of products exhibited mono-demethylated cap structures (m^7^GpppN or GpppNm). Since mass measurement alone cannot pinpoint the exact site of methylation loss, this finding highlights the necessity for stringent quality control of raw materials in co-transcriptional capping workflows to prevent impurity generation at the source. Future studies will focus on elucidating key process parameters influencing mRNA capping efficiency (e.g., cap analog feed ratio, effects of T7 RNA polymerase mutants, enzymatic reaction kinetics, and thermal stability of the cap structure). Additionally, we will explore the expanded application of this method in continuous manufacturing monitoring and comprehensive multi-component impurity profiling, thereby providing robust theoretical and technical support for the quality consistency evaluation and continuous process optimization of mRNA vaccines.

## 5. Conclusions

In conclusion, this study establishes a streamlined, high-throughput LC-MS assay for the precise quantification of mRNA capping efficiency. By optimizing guide DNA probes, RNase H cleavage specificity was enhanced to ≥98% with high conversion efficiency, outperforming DNAzyme-based approaches. The use of thermostable RNase H facilitates a unified workflow by merging denaturation and site-directed cleavage, significantly reducing sample processing complexity. On the chromatographic side, strategic optimization of the ion-pairing reagent system effectively suppressed metal adduct formation during ESI-MS, reducing total adduct abundance to <10%. This advancement permits direct injection of 5′ cap fragments without prior purification. Collectively, these innovations reduce the total analytical workflow from 4–6 h to under 1.5 h. Comprehensive cross-platform validation confirms the method’s independence from specific hardware and proprietary software algorithms, demonstrating robust quantitative accuracy across a wide capping efficiency range (50–99%). Real-world application to diverse mRNA vaccine bulk materials further validates its universal applicability and reliability for batch release testing. Critically, this approach overcomes key limitations of conventional techniques by eliminating the cumbersome sample preparation and labor-intensive purification steps inherent to traditional LC-MS workflows. By delivering a platform-independent, scalable, and universally adaptable analytical strategy, this method provides a practical and efficient solution for the quality control of mRNA therapeutics. It is well-positioned to serve as a useful tool for process development, impurity profiling, and stability monitoring in the rapidly expanding mRNA vaccine and therapeutic landscape.

## Figures and Tables

**Table 1:** Recovery rates of various fragments under different pretreatment methods.

Test Parameters	Theoretical Cap Value%	98.54	94.54	89.54	69.54	49.54
	Theoretical Uncap Value%	1.46	5.46	10.46	30.46	50.46
15 nt fragment	Measured Uncap %	1.91	4.83	8.08	25.13	46.33
	RSD (*n* = 3)	3.63	7.52	7.19	1.67	0.43
	Mean Recovery%	127.33	87.82	76.95	82.39	91.74
15 nt fragment treat with CIAP	Measured Uncap%	1.44	4.09	8.73	27.72	47.03
	RSD (*n* = 3)	24.22	9.20	0.50	0.38	0.41
	Mean Recovery%	98.40	74.91	83.46	91.00	93.20
25 nt fragment	Measured Uncap%	1.44	5.37	10.10	31.05	50.96
	RSD (*n* = 3)	2.13	2.33	0.98	0.83	0.52
	Mean Recovery%	98.40	98.35	96.56	101.94	100.98
25 nt fragment treat with CIAP	Measured Uncap%	1.34	5.52	10.29	31.20	50.11
	RSD (*n* = 3)	5.07	2.33	0.34	0.30	0.42
	Mean Recovery%	96.88	102.52	99.07	102.67	99.45

**Table 2:** Intra-day and inter-day precision data for the method.

Cap1 Value		Conventional Level %		Near Threshold Level %	
Day Time		Day 1	Day 2	Day 1	Day 2
Experimenter 1	repeat1	95.86	94.45	88.93	88.51
	repeat2	95.92	95.25	89.00	88.15
	repeat3	95.67	94.82	87.80	88.07
Experimenter 2	repeat4	95.60	96.36	87.70	88.27
	repeat5	95.60	96.41	87.61	88.25
	repeat6	95.48	96.56	87.58	88.28
Mean %		95.69	95.64	88.10	88.25
Intra-day RSD%		0.18	0.96	0.76	0.17
Inter-day RSD%		0.66		0.53	

**Table 3:** Quantification of capped, uncapped, and cap-degraded mRNA across multiple manufacturers (ND indicates not detected).

Manufacturers	Capping Method	Batchs	Cap1	Cap0	G Cap	Uncap	Cap1 Degraded
1	Enzymatic capping	Batch1	99.73%	0.27%	ND	ND	ND
		Batch2	99.63%	0.37%	ND	ND	ND
		Batch3	99.68%	0.32%	ND	ND	ND
2	Enzymatic capping	Batch1	98.93%	0.61%	0.22%	0.18%	0.06%
		Batch2	98.65%	0.76%	0.24%	0.30%	0.05%
		Batch3	98.79%	0.58%	0.29%	0.28%	0.06%
3	co-transcriptional capping	Batch1	96.10%	ND	ND	1.54%	2.36%
		Batch2	96.02%	ND	ND	1.65%	2.33%
		Batch3	96.88%	ND	ND	1.33%	1.79%
4	co-transcriptional capping	Batch1	97.68%	ND	ND	1.74%	0.58%
		Batch2	97.57%	ND	ND	2.07%	0.36%
		Batch3	97.35%	ND	ND	2.11%	0.54%
5	co-transcriptional capping	Batch1	95.54%	1.52%	ND	0.09%	2.85%
		Batch2	96.38%	1.00%	ND	ND	2.62%
		Batch3	96.33%	0.93%	ND	0.13%	2.61%
6	co-transcriptional capping	Batch1	94.49%	ND	ND	4.80%	0.71%
		Batch2	94.52%	ND	ND	4.60%	0.88%
		Batch3	94.15%	ND	ND	5.14%	0.71%
7	co-transcriptional capping	Batch1	98.94%	ND	ND	0.82%	0.24%
		Batch2	98.85%	ND	ND	0.92%	0.23%

## Data Availability

All data from the study have been included in the manuscript itself.
